# Systematic review of available software for multi-arm multi-stage and platform clinical trial design

**DOI:** 10.1186/s13063-021-05130-x

**Published:** 2021-03-04

**Authors:** Elias Laurin Meyer, Peter Mesenbrink, Tobias Mielke, Tom Parke, Daniel Evans, Franz König, on behalf of EU-PEARL (EU Patient-cEntric clinicAl tRial pLatforms) Consortium

**Affiliations:** 1 Center for Medical Statistics, Informatics, and Intelligent Systems, Medical University of Vienna, Vienna, Austria; 2 Novartis Pharmaceuticals Corporation, One Health Plaza, East Hanover, NJ USA; 3 Janssen Cilag GmbH, Neuss, Germany; 4 Berry Consultants, Abingdon, UK; 5 Pfizer R&D UK Ltd, Sandwich, UK

## Abstract

**Background:**

In recent years, the popularity of multi-arm multi-stage, seamless adaptive, and platform trials has increased. However, many design-related questions and questions regarding which operating characteristics should be evaluated to determine the potential performance of a specific trial design remain and are often further complicated by the complexity of such trial designs.

**Methods:**

A systematic search was conducted to review existing software for the design of platform trials, whereby multi-arm multi-stage trials were also included. The results of this search are reported both on the literature level and the software level, highlighting the software judged to be particularly useful.

**Results:**

In recent years, many highly specialized software packages targeting single design elements on platform studies have been released. Only a few of the developed software packages provide extensive design flexibility, at the cost of limited access due to being commercial or not being usable as out-of-the-box solutions.

**Conclusions:**

We believe that both an open-source modular software similar to OCTOPUS and a collaborative effort will be necessary to create software that takes advantage of and investigates the impact of all the flexibility that platform trials potentially provide.

**Supplementary Information:**

The online version contains supplementary material available at 10.1186/s13063-021-05130-x.

## Introduction

**Fig. 1:**
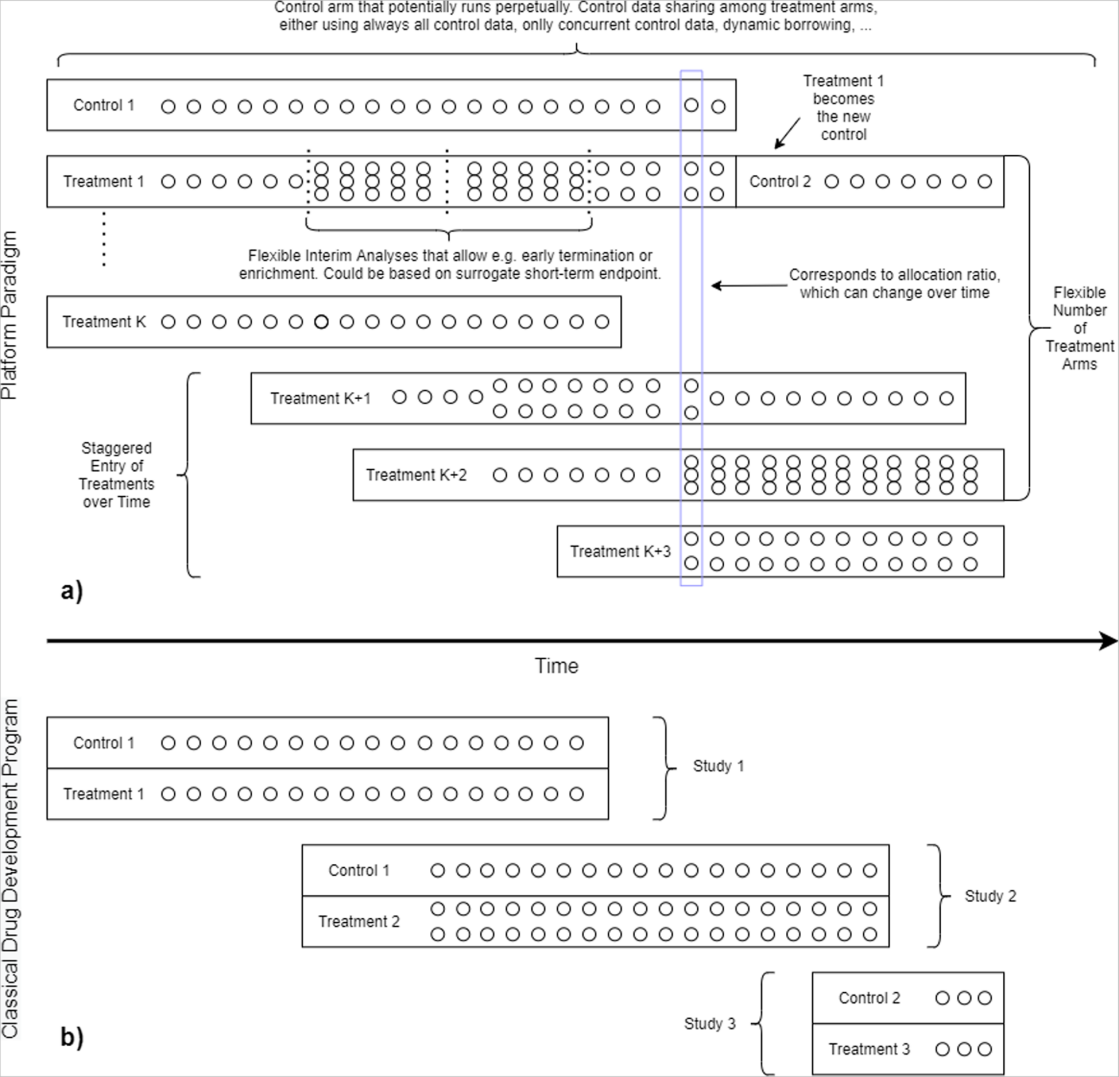
Comparison of platform paradigm (**a**) and classical drug development program (**b**). Many features can be included in the platform paradigm which are usually not found in classical drug development programs consisting of sequential/parallel two-arm studies, such as a flexible number of interim analyses (possibly using short-term surrogate endpoints), adaptive randomization ratios, a staggered entry of (a flexible number of) treatments over time, change of control treatment within the trial, and different control arm data sharing options

Master protocol trials allow for the evaluation of both multiple investigational treatments and multiple subgroups of the study population within the same overall clinical trial structure, as compared to traditional randomized controlled trials, where usually only one investigational treatment is investigated in one study population [1]. Several types of master protocol trials can be distinguished, such as basket trials, umbrella trials, and platform trials. Whereas in classical development programs different studies are needed for newly available treatments, adaptive platform trials are a type of randomized clinical study that allow for the evaluation of multiple interventions in a disease or condition in a perpetual manner, with interventions entering and leaving the platform on the basis of a predefined decision algorithm (definition following the Adaptive Platform Trials Coalition [2]). Figure 1 illustrates the difference between the platform paradigm and a classical drug development program. One of the major advantages of platform trials is their reduced sample size due to the sharing of a common control arm. The platform trial design offers other important potential advantages compared to the traditional approach of running many studies either sequentially or in parallel, including an overall reduction in the trial infrastructure and the removal of competition between trials within a limited pool of patients. These trial designs are increasingly gaining attention and popularity and are considered in many disease areas to facilitate the evaluation of new or targeted therapies, clinically validated targets and predictive biomarkers, and faster clinical testing of available compounds. Many recent reviews have focused on both planned and conducted platform trials and other types of master protocol trials, including their related methodology, highlighting the potential advantages and disadvantages of such trial designs [3–6]. However, no special focus has been given to identifying software relevant to the design of such trials. A recent overview highlights the most important design concepts of platform trials, most of which also pose the greatest statistical and operational challenges [4]. These challenges include whether or not potential control data will be shared across treatment arms (leading to potentially non-concurrent control data), allocation ratios, rules and mechanisms for adding and dropping of treatment arms over time, timing, decision rules (Bayesian or frequentist) and endpoints for interim and final analyses and mechanisms of stopping the platform trial. Multi-arm multi-stage designs (MAMS) have been recommended in the literature to compare several treatments allowing for potentially several interim analyses. For example, in rare diseases, sharing a common control group helps to reduce the sample size as it may not be practical or feasible to conduct multiple placebo-controlled trials. Since multiplicity correction across treatments is often not needed or planned [7–10], software aimed at designing MAMS trials is of immediate interest to designing platform trials. Many reviews of software available for designing adaptive and group sequential trials are available [11, 12], most notably a recent comprehensive review [13]; however, no review so far has assessed software with the particular aim of designing platform trials.

In this systematic review, a comprehensive overview is provided of available software (both free and commercial), which is relevant for the simulation of platform trials, i.e., this includes simulation for platform trials, umbrella trials, multi-arm and/or multi-stage trials, and software relevant to adaptive designs in general.

## Methods

**Fig. 2:**
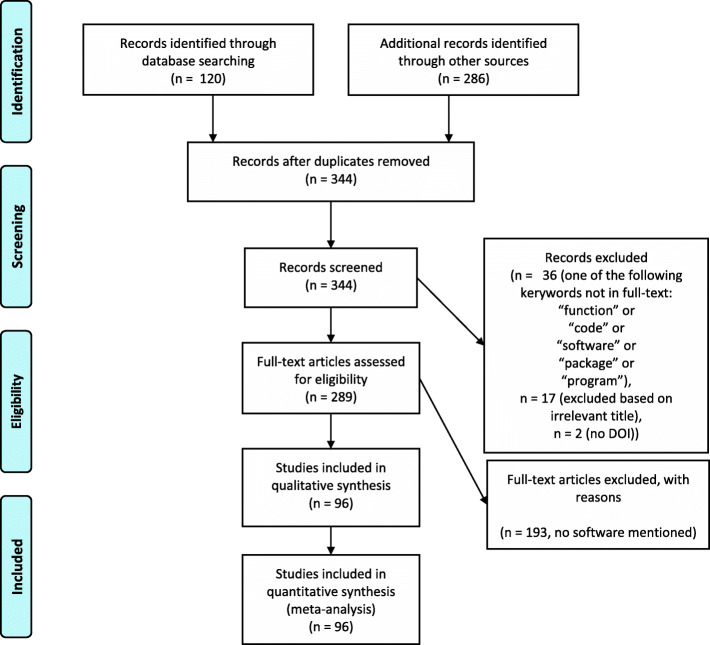
PRISMA flow diagram of systematic article selection process

This study was conducted in accordance with the PRISMA reporting guidelines for systematic literature reviews [14] (PRISMA checklist in supplements and PRISMA flowchart in Fig. 2).

### Data sources and searches

We conducted a systematic literature search on Scopus ^R^ and the Web of Science Core Collection (from inception to 29.05.2020) searching for the following terms in the title, abstract or keywords: ““simulation” + (“platform trial*” OR “platform stud*” OR “platform design*” OR “umbrella trial*” OR “umbrella stud*” OR “umbrella design*” OR “multi-arm trial*” OR “multi-arm stud*” OR “multi-arm design*” OR “multi-stage trial*” OR “multi-stage stud*” OR “multi-stage design*”) + “clinical””. We further considered all identified papers from a recently published systematic literature review on master protocol trials which used similar search terms [3]. In addition, we recorded relevant software identified by other software reviews mentioned previously [11–13] and through manual online searches using appropriate search terms similar to the literature search terms (e.g., on https://rseek.org/, which is basically a search engine filtering R-related content, and https://github.com/).

### Study selection

Only articles in English were considered. As a first step in the screening process, titles were assessed for relevancy. Subsequently, existence of a DOI was checked. Finally, full-texts were pulled and, using software, checked for the presence of one of the keywords: “code,” “software,” “package,” “program,” or “function.” Remaining papers were eligible for full-text assessment by the authors. After a pilot phase, in which ELM assessed 10 of the remaining papers, papers were randomly distributed among co-authors for full-text assessment, such that in total ELM assessed 49 papers and the rest of the authors 48 each. In case of uncertainty, the full text was again assessed by ELM and FK.

### Data extraction

For each paper, in addition to standard metrics such as journal, year of publication, etc., we recorded whether any software was mentioned or provided and whether it was relevant for the simulation of platform trials. The full list is included in the supplements S1. For relevant software, we recorded whether they satisfied key features of platform trial simulation such as facilitating staggered entry of treatments over time or allowing different options regarding data sharing.

### Data synthesis

Quantitative and qualitative synthesis of papers is presented in the “Results” section. Software of particular interest was investigated further and a summary can be found in Table 2.

## Results

**Fig. 3:**
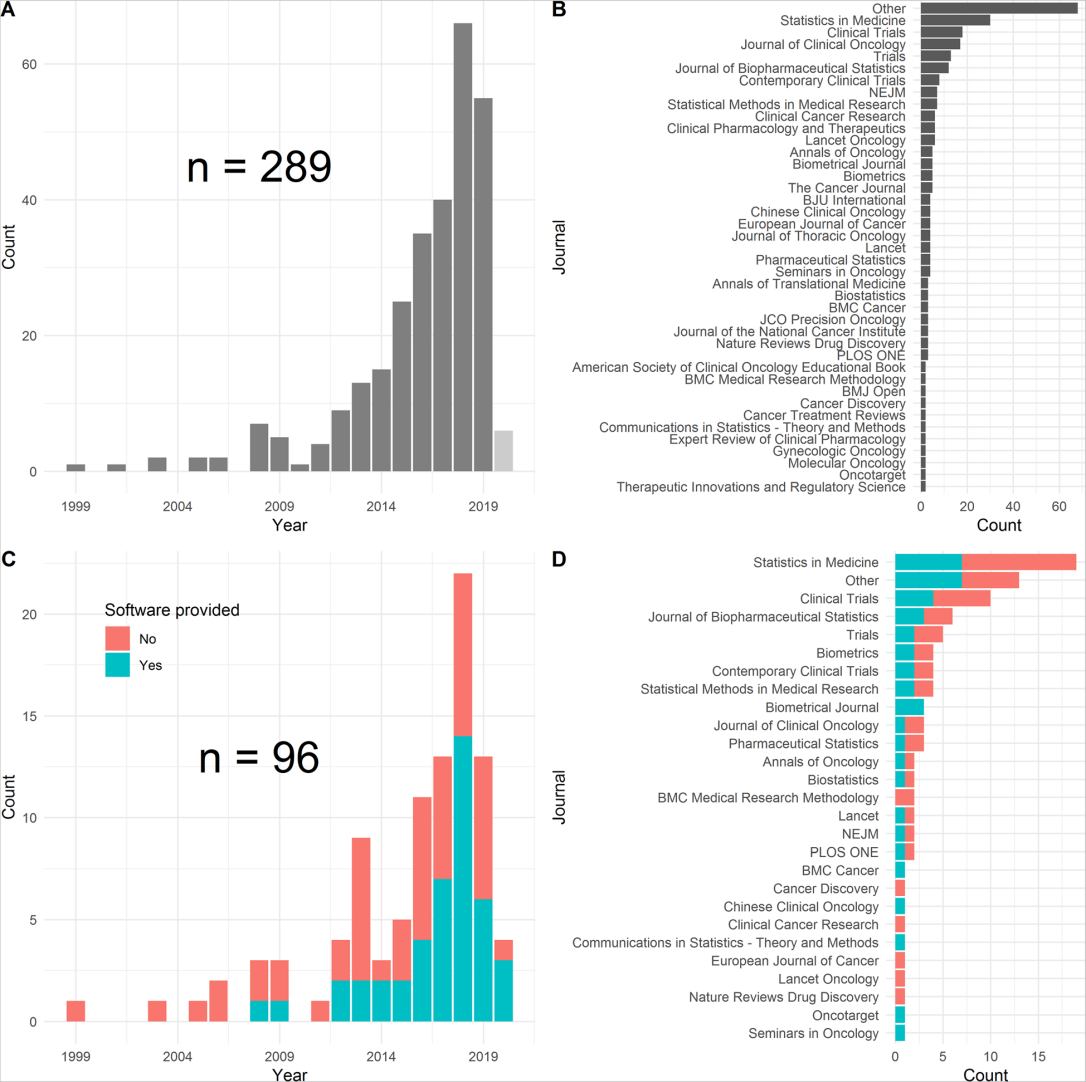
**a** Year of publication of identified articles. Please note that for 2020, only papers up to 29.05.2020 which were found on Scopus and Web of Science Core Collection with the search terms described in the “Methods” section were included. **b** Journal of publication of identified articles. Journals having published only one article were summarized as “Other”. Journals are sorted firstly from highest to lowest number of publications and later alphabetically. **c** Year of publication of identified articles which mentioned any software. Please note that for2020 only papers up to 29.05.2020 which were found on Scopus and Web of Science Core Collection with the search terms described in the “Methods” section were included. Colors represent whether or not any software was provided in the paper either as a supplement or direct link to some code repository. **d**: Journal of publication of identified articles which mentioned any software. Journals having published only one article were summarized as “Other”. Journals are sorted firstly from highest to lowest number of publications and later alphabetically

In total, 289 full-text papers were assessed (for an overview of the screening process, see the PRISMA flowchart in Fig. 2). We found that 96 papers (33%) mentioned software (not necessarily related to platform trial simulation) and in 44 papers (15%) software was provided, which is very similar to the findings of Grayling and Wheeler [13], who also found that only 30% of their included articles made their code available in some form. Of the papers mentioning any software, 32% (31/96) mentioned software which we considered relevant and 40% (38/96) mentioned software which we considered to be of secondary interest, such as code snippets for a particular task which could be incorporated in simulation software. Figure 3 shows the distribution of identified articles with respect to year of publication and the number of identified articles with respect to publishing journal, for both all 289 papers assessed and the subset of 96 papers which mentioned any software. We can see an exponential increase in papers up to 2018/2019, for both all assessed papers and the subset mentioning software. In terms of publishing journals, we observed that software was mentioned in applied journals to discuss the operating characteristics of real platform trials such as STAMPEDE [15]. Software is primarily mentioned in applied statistical journals such as Statistics in Medicine where software (code) is mentioned in context of evaluating the operating characteristics of proposed design and analysis methods. In many of the methodological journals, the software was referenced without including in the supplementary materials or appendix nor with a link to the website where the software is available. One exception is the Biometrical Journal, where software code must be provided due to its reproducible research policy. In this journal, a software check is implemented so that results presented in the paper can be easily reproduced. Furthermore, we noted many simulation studies that provided results, but neither mentioned nor provided software (code), which is a finding similar to Grayling and Wheeler [13].

The review highlighted that software developed in the context of adaptive and group sequential designs may also be (partially) applied in the context of platform trials. Software for adaptive designs cover many aspects of platform trials allowing for multiple interim analyses, multiple treatment arms (adaptive seamless designs) or subgroup selection (adaptive enrichment designs). For example, if there is no multiplicity adjustment across treatment arms, then standard group sequential boundaries might be used if one wants to adjust for repeated significance testing within each treatment (or treatment-control comparison). Adaptive design software developed in the context of multi-armed clinical trials with interim analysis might be applicable with the restriction that treatment arms usually are fixed in the beginning of the trial allowing only selection/dropping of treatments at interim analyses. Usually, the staggered entry of treatments is not foreseen, but the use of such software may still be of interest to describe the operational characteristics and determine reasonable sample sizes for the initial set of treatments to start a multi-armed trial. Possible group sequential and adaptive design programs include standalone software such as EAST or ADDPLAN and R and Stata packages such as asd and nstage. We acknowledge that this paper does not include a full review of all adaptive and group sequential software which could be similarly applied and refer to Bauer et al. [11], Grayling and Wheeler [13], and the appendix of Wassmer and Brannath [12] for recent reviews of more adaptive software. For an overview of systematically identified software which could potentially be adapted to be useful in the context of platform trial simulation, see Table 1. In total, we identified 9 software solutions of immediate interest to the design and simulation of platform trials. All the identified software of immediate interest is described verbally in the following section, whereby we differentiate standalone software, software packages based on R and Stata which need to be run within their respective programming environments and online trial simulators. A summary of features is provided in Table 2.

**Table 1:** Additional non-commercial software of potential interest to platform trial simulation. Description of most R packages was taken from https://cran.r-project.org/web/packages/. All URLs were checked last on 29.07.2020

Name	Description	Platform
adaptTest	The functions defined in this program serve for implementing adaptive two-stage tests. Currently, four tests are included: Bauer and Koehne (1994), Lehmacher and Wassmer (1999), Vandemeulebroecke (2006), and the horizontal conditional error function.	R
AGSDest	Calculation of repeated confidence intervals as well as confidence intervals based on the stage-wise ordering in group sequential designs and adaptive group sequential designs.	R
basket	Package for the analysis of basket trials which implements binary, symmetric multi-source exchangeability models for Bayesian analyses of subgroups. Analyses can be conducted “basketwise” or “clusterwise”, where subgroups are combined into meta-baskets.	R
BayesianPickWinner	Calculation of power for the Bayesian pick-the-winner design. Accessible via https://github.com/dungtsa/BayesianPickWinner.	R
Bayesian Basket Discovery Trials	Design, Monitoring and Analysis. Accessible via https://brbnci.shinyapps.io/BasketTrials/	R Shiny
BTcode	Basket trials with hierarchical Bayesian modeling. Accessible via: https://github.com/kristenmay206/BTcode	R
EVD	Simulation of various sequential two- and multi-arm clinical trial designs with Day-14 survival rate as primary endpoint. Accessible via https://github.com/mbrueckner/evd	R
gMCP	Functions and a graphical user interface for graphical described multiple test procedures.	R
interAdapt	A shiny application for designing adaptive clinical trials. For more details, see: http://arxiv.org/abs/1404.0734	R Shiny
MD Anderson Software Download Kiosk	Several individual software for specific tasks, such as software to simulate clinical trials with response-adaptive randomization (no control, multiple experimental arms) and a tool to calculate confidence limits on the difference between two binomial proportions. Accessible via https://biostatistics.mdanderson.org/SoftwareDownload/.	Individual
multcomp	Simultaneous tests and confidence intervals for general linear hypotheses in parametric models, including linear, generalized linear, linear mixed effects, and survival models.	R
optGS	Near-optimal and balanced group-sequential designs for clinical trials with continuous outcomes	R
Optimal two-stage basket trials	Calculation of optimal design parameters for two-stage basket trials. Accessible via https://www.mskcc.org/departments/epidemiology-biostatistics/biostatistics/basket-trials.	R
RCTDesign	Evaluating, analyzing, and reporting group sequential and adaptive clinical trial designs.	R
Research code of Dr. James, Wason	Code in several programming languages implementing research of Dr. James Wason from the MRC Biostatistics Unit and co-authors. Accessible via https://sites.google.com/site/jmswason/supplementary-material	Several
Research code of Dr. Ying Yuan	R code and Windows programs implementing research of Dr. Ying Yuan from the MD Anderson Cancer Center. Accessible via https://odin.mdacc.tmc.edu/~yyuan/index_code.html	R and Individual

**Table 2:** Summary of features of identified software of immediate interest. A checkmark represents an available feature, a checkmark in parentheses represents a partly available feature, a wave stands for a feature for which it is foreseen that the user includes it by writing additional code and an x represents an unavailable feature. Software is grouped into standalone software, packages for R and Stata and online trial simulators. MD Anderson is the abbreviation of Integrated Platform for Designing Clinical Trials

Feature	Description	FACTS	ADDPLAN	EAST	OCTOPUS	nstage	MAMS	asd	HECT	MD Anderson
Flexibility arms	Options regarding the number of treatment arms at the start of the trial	✔	✔	✔	✔	✔	✔	✔	✔	✔
Staggered entry	Options regarding the staggered entry of treatments over time, such as pre-planned, randomly, replacing treatments, ...	x	x	x	∼	x	x	x	x	(✔)
Endpoints	Choice, e.g., binary, continuous, time-to-event	✔	✔	✔	✔	✔	✔	✔	✔	x
Surrogate endpoint	Option to use different endpoint at interim	✔	(✔)	x	∼	✔	✔	✔	✔	x
Interims	Options regarding the number and timing of interim analyses	✔	✔	✔	✔	✔	✔	x	✔	✔
Enrichment/seamless	Dedicated options for treatment selection or enrichment at interim	✔	✔	✔	∼	x	x	✔	x	x
Bayesian	Bayesian decision rules	✔	x	x	✔	x	x	x	✔	✔
Frequentist	Frequentist decision rules	✔	✔	✔	∼	✔	✔	✔	x	x
Flexibility data sharing	Advanced options for data sharing, such as sharing control data, sharing only concurrent control data, dynamic borrowing, etc.	x	x	x	(✔)	x	x	x	x	x
Multiplicity correction	Options for multiplicity correction, e.g., control of FWER, FDR, ...	x	✔	✔	∼	(✔)	✔	✔	x	x
Flexibility allocation ratio	Options regarding the allocation ratio of patients to the arms	✔	(✔)	(✔)	∼	✔	✔	x	(✔)	✔
RAR	Response-adaptive randomization	✔	x	x	∼	x	x	x	✔	✔
Flexibility cohort structure	Options for designing exact cohort structure to enter/leave platform, e.g., always control and two experimentals, ...	x	x	x	x	x	x	x	x	x
Flexibility recruitment rate	Options regarding the recruitment rate of patients to the trial	✔	(✔)	✔	✔	✔	x	x	x	x
Comparison classical development program	Built-in options regarding the comparison with more classical designs	x	x	x	x	x	x	x	(✔)	x
GUI	Graphical user interface	✔	✔	✔	x	✔	x	x	✔	✔
Commercial	Software is commercial	✔	✔	✔	x	x	x	x	x	x
Open Source	Open source code available	x	x	x	✔	✔	✔	✔	✔	x

### Standalone software

#### FACTS

FACTS [16] is a commercial software package developed and maintained by Berry Consultants.

The FACTS software (the “Fixed and Adaptive Clinical Trial Simulator”) simulates clinical trials in order to facilitate the design of the statistical analysis. One key feature of the package is the estimation of a design’s operating characteristics where closed form analysis is not possible, but also to estimate additional operating characteristics, and to take into account operational issues such as time to endpoints, accrual rates, and dropout rates. The FACTS simulator is grouped into different ‘classes’ of trials (currently: dose escalation, treatment arm comparison, population sub-group testing, and a sequence of arm comparison trials) and within each class a simulator is provided for each supported endpoint type (currently: continuous, dichotomous or time-to-event). Within these classes, FACTS can simulate “basket” and “umbrella” trials including the use of interims, dropping of sub-groups or treatments, and the use of longitudinal modeling to improve early decision making when there is a long time to subjects’ final data. A simulator for Platform Trials is in development.

Each simulator is a closed, command line program implemented in C++, these simulators execute the simulations extremely quickly. There is no opportunity, or requirement for the user to do any programming or write any code or expressions. Everything is by selection of options and the specification of parameter values. To assist in this selection and specification, there is a unified user interface program that helps the user with the selection and specification of the required parameters, runs the simulations and displays the simulation results.

FACTS differs from most other trial design software in that it is first and foremost a simulator, rather than a statistical analysis package that uses simulation for some specific numerical integration. Secondly rather than providing a collection of different programs for different designs, within each class of trial, it provides a wide range of inter-operable options from which a wide range of different designs can be created. This allows FACTS to be used by the biostatistician in an iterative and creative fashion with the clinical team, adding and refining design options and sharing simulation results at every stage.

#### ADDPLAN

ADDPLAN [17] is statistical design, simulation and analysis software, which was originally developed with the primary focus on confirmatory adaptive group-sequential designs. ADDPLAN is comprised of four modules: BASE, MC (multiple comparisons), PE (population enrichment) and DF (dose finding). All modules focus on frequentist methodology. As a result, large numbers of study design simulations may be completed in a short time. ADDPLAN BASE, MC, and PE allow the design of studies including early futility and efficacy stopping, sample size re-estimation, treatment selection, and population enrichment. ADDPLAN DF supports the design of dose-escalation studies and fixed and adaptive MCPMod Dose Finding designs, putting particular emphasis on adaptive randomization procedures. ADDPLAN MC might be of particular interest for the design of platform studies, as it allows to simulate multi-armed studies with common control. However, the software has not been optimized for the purpose of simulating platform studies with staggered entry of treatment arms or using concurrent controls. It is generally assumed in ADDPLAN MC that a study starts to enroll in all treatment arms simultaneously, while dropping of arms may occur at one specific interim analysis only. All adaptions are based on the primary endpoint. Only survival endpoints permit within ADDPLAN the use of a surrogate for decision making in an interim analysis. Operational aspects of study designs cannot be simulated within ADDPLAN, and post-processing of simulation results may be required to understand the operational implications of study design challenges, including timing of interim analyses and number of enrolled patients vs. number of patients with primary endpoint available. ADDPLAN has long been a stand-alone software without any open API, such that its subroutines could not be used in R (or other software), while ADDPLAN was also not able to execute any external software within simulations or analyses (like R). However, the latest version of ADDPLAN (ADDPLAN NEO) allows the execution of R code within simulations and analyses, thereby possibly allowing for additional extensions to simulate platform studies.

#### EAST

EAST [18] is statistical software for the design, simulation, and monitoring of adaptive, group sequential and fixed sample size trials. EAST is comprised of a base module, as well as several add-on modules, such as Exact (binomial response trials using exact distributional assumptions), Endpoints (multiple endpoints), and Sequential (group sequential clinical trial designs). Two modules of particular interest in the context of platform trials are MAMS and Enrich. MAMS is an EAST module that facilitates design and monitoring of multi-arm multi-stage studies with options for dose selection, sample size re-estimation, and early stopping. It implements group sequential theory extended for multi-arm setup [19] and multi-arm two-stage designs with normal and binomial endpoints using a *p* value combination approach [20]. Enrich is an EAST module that facilitates the ability to simulate a population enrichment design with a survival endpoint. It starts with two populations and allows the user to decide based on an interim analysis whether to continue enrolment in both subpopulations with or without sample size re-estimation, only continue enrollment from the sub-population of interest, or terminate the trial for futility.

### Packages for R and Stata

#### OCTOPUS

OCTOPUS [21] is an R package which has been developed with the objective to help drug developers simulate platform trial designs. OCTOPUS can be readily utilized as a tool simulating basic platform trials including various design options, endpoints, and operational scenarios. A “CreateProject” function eases the development of a platform design and simulation scenarios from a number of generated template R-files, implementing the functions, which might typically require further customization. Extensive documentation and example projects are available on GitHub [21]. OCTOPUS can be interpreted as an R-platform to build and run platform simulations. Simulations in OCTOPUS are comprised basically of four blocks: operation simulation, data simulation, data analysis, and simulation output. Each of these blocks can be adjusted by either using functions currently available (in OCTOPUS) or adding new functions, allowing for a high level of customization. While the “CreateProject” function and other available implemented functions of OCTOPUS enable the rapid development of simulations, an even greater benefit of OCTOPUS is the ability to customize design options and the potential to include alternative R packages with their available analysis code (e.g., DoseFinding). The template R-files developed in the.

“CreateProject” command contain simple functions which may be customized for the specific analyses as required by the user. Deeper-level adjustments to methods implemented in OCTOPUS can be applied using the S3 class of generic methods. For this purpose, a good understanding of the OCTOPUS package structure, code and functional variables is typically required. Without this good understanding, a lot of time may easily be spent on debugging the own implementations to make it fit into OCTOPUS. OCTOPUS is available via GitHub and is still in continued development.

#### nstage Stata module

Bratton et al. [22] presented an update to their menu-driven Stata program nstage, which can be used to design multi-arm multi-stage (MAMS) trials with time-to-event endpoints. Options include the number of stages, accrual rate, pair-wise significance level, pair-wise power, number of arms at each stage, hazard ratios, correlation between hazard ratios of interim and final endpoints, and many more. The output provided includes information on operating characteristics for each stage (power, type 1 error, patients, time, etc.), as well as overall pair-wise and family-wise type 1 error estimates. nstage was used to design the STAMPEDE [23] and FOCUS4 [24] trials. An extension for binary endpoints, nstagebin, exists.

#### MAMS R package

Jaki et al. [25] introduced the R package MAMS, which facilitates design of MAMS trials with normal, binary, ordinal or time-to-event endpoints within the group-sequential framework. In terms of design options, the user can specify the number of treatments, stages, treatment effects, power, type 1 error, randomization ratio, types of boundaries and also whether only the most promising or all promising treatments should be continued after interim. In contrast to the nstage Stata module, the focus is on controlling the family-wise error rate in the strong sense. A dedicated function is available which takes a given study design and computes several operating characteristics, such as the probability of rejecting at least one hypothesis and the expected sample size, but also allows the specification of particular hypotheses for which the probability of rejection should be computed.

#### asd R package

Parsons et al. [26] introduced the asd R package in 2011 as a simulation platform for adaptive seamless phase II/III trials, whereby treatment or dose selection at interim analysis is based on either a short-term or the final normally distributed outcome. The power and type 1 error for any combination of the hypotheses can be computed. Over time, the package was enhanced, such that now it facilitates multi-arm two-stage treatment selection designs, more outcome measures and subgroup selection designs. Two example studies that use the asd package for planning can be found in Friede et al. [27].

### Online trial simulators

#### HECT

The “Highly Efficient Clinical Trials Simulator (HECT): Software application for planning and simulating platform adaptive trials” [28] is an R Shiny app accessible at https://mtek.shinyapps.io/hect/. This software tool facilitates the simulation of platform trials with up to 10 treatment arms, whereby treatment arms can either be compared to each other or against a reference treatment. Both continuous and binary endpoints can be chosen. Arms can be dropped and graduated at specified interim analyses based on Bayesian posterior probabilities; however, few options exist for new treatment arms to enter the trial over time. When trials are compared against each other, response-adaptive randomization can be implemented after a certain burn-in period. Detailed information for single trial simulations is provided, as well estimated type 1 error and power, whereby the definition of these operating characteristics depends on the scenario. For these operating characteristics, a comparison with an RCT with fixed sample size, no interim analyses and balanced randomization is provided as well. More information can be found in the R Shiny app under the “User Manual” tab.

#### Integrated Platform for Designing Clinical Trials

Accessible on https://trialdesign.org/, this software catalog by researchers from the MD Anderson Cancer Center offers more than 25 different programs for clinical trial design, ranging from sample size calculation to simulating basket and platform trials. In terms of platform trials, two programs stand out: (1) An implementation of a Bayesian drug combination platform trial design with adaptive shrinkage [29] and (2) an implementation of a Bayesian platform design using adaptive randomization with early futility and/or efficacy stopping, which allows multiple arms with or without a control group, whereby decisions are based on posterior probabilities and the endpoint is binary. The latter provides a range of simulation settings to be chosen such as the number of active arms at all times, the number of maximum arms overall, prior distributions for all arms, maximum sample size overall and per arm, randomization types, and Bayesian decision rules. Whenever an arm stops, it is replaced by another arm, as long as the maximum number of arms is not exceeded. Patient outcomes, the trial timeline, efficacy and futility stopping and randomization probabilities can be monitored; however, it seems that no option is available to compute operating characteristics.

## Suggestions for future platform trial design software

The potential design options implemented in platform trials are almost infinite, which explains the limitations of broadly available software to design, simulate, and analyze platform trials (apart from the novelty of the design approach in general). Software which is developed based on project needs is typically limited in the variety of available design options for comparison, as such software is developed for a particular need, not for researching all potential new approaches to clinical research and statistical science. On the other hand, software solutions, which allow for a wide range of design options, may easily overload the user with requirements for design specifications. In the end, experienced modelers and simulation programmers may find it more efficient to write their own simulation code rather than starting to deep-dive into the required specifications for a new flexible software. These considerations lead to two potential software development trajectories:

- Development of many stand-alone packages each of which is focused on specific platform trial designs, but being limited in their flexibility for design customization
- Development of a common platform-software-platform, which allows one to combine different required design elements in a simple manner

Obviously, a well-designed and implemented platform-software-platform will be able to also handle the standalone packages. OCTOPUS has been named in this paper as one candidate for such a platform. It is still a young package, which allows for a high level of flexibility in study design customization via the implemented S3 class functions. However, customization of study designs requires extensive understanding of the object structures and the simulation and analysis architecture. Alternatively, standalone packages for specific platform designs could allow for wider customization by inclusion of generic functions, which would provide the user with the ability to submit their own function implementations. Obviously, the standalone packages would then become closer to the aforementioned platform-software-platform.

A project to develop a platform-software-platform could collapse under its own complexity if it tries to provide all options from the outset, and may well find that it has wasted time on providing options that turn out to be rarely required. Thus, it needs to start with a fairly limited scope. The standalone packages on the other hand can include complexities that need to be evaluated for their particular trial, and the accumulation of experience of “what works” subsequently informs the platform-software-platform. For a summary of features required in platform simulation software, see Table 3. We differentiate between “core” and “prospective” features, whereby the “core” features should be included in any platform trial simulation software, as it contains to a large extent the main features that distinguish a platform trial from more classical development programs (see Fig. 1). Such features include different options for control data sharing and staggered entry of treatments over time, both of which are barely covered by existing software (see Table 2). The “prospective” features are features that are not necessary for a basic simulation application but will be required to properly model the complexities of a platform trial and should therefore be kept in mind when programming the software.

**Table 3:** Overview of features for platform trial simulation software. We differentiate two programming steps: the base features necessary for the majority of platform trials (Core) and features that may be necessary for certain platform trials, but will not be necessary for all, or even a majority of platform trials (Prospective). These features do not need to be part of the core, or initial software package, but the software should be constructed with the perspective that these may become required extensions to the simulation software in the future. We furthermore differentiate features that belong to active investigator design choices (Design), features that belong to investigators’ assumptions about the reality at the design stage (Assumption) and features that belong to essential simulation information that need to be reported (Reporting)

Step	Type	Feature
Core	Assumption	Simulate the availability and timing of arrival of new treatments
Core	Assumption	Specify the response of the treatment - this might be the specific response of each treatment, or the distribution(s) of possible treatment response to sample from
Core	Design	Number of active arms at start and maximum number of active arms throughout trial
Core	Design	Specify the allocation between treatments and control, possibly varying with the number of treatments in the trial (ideally including option for response-adaptive randomization)
Core	Design	Different options for control arm data sharing - either comparison with all control subjects or only contemporaneous subjects
Core	Design	Specify interim timings - either a trial sequence of interim analyses, or per-treatment sequence
Core	Design	Specification of interim (early success/futility/enrichment) and final decision rules (frequentist or Bayesian)
Core	Design	Specify “full information” rules for when a treatment completes
Core	Design	Specify platform stopping rules (e.g., Maximum time, number of subjects or number of treatments)
Core	Design	Rules to cater for times when a) there is no treatment in the trial and b) there is only one treatment in the trial
Core	Reporting	Reporting of time to find first success, the number of treatments tested to find the first success, the number of subjects tested to find the first success, the number of subjects on control to first success
Core	Reporting	Reporting of proportion of treatments with a target response or better that are successful, the proportion of treatments with the same response as control that are successful, applicable error rates and power concepts
Core	Reporting	Built-in comparison with comparable classical development program to evaluate efficiency gains, which will depend on the trial under investigation (i.e., is it a phase 2a/2b or 2b/3)
Prospective	Assumption	Correlation between surrogate endpoint and final endpoint
Prospective	Design	Ability to simulate a surrogate endpoint for interim decisions or early visit data of the final endpoint
Prospective	Design	Ability to choose type of endpoint (binary, continuous, time-to-event, ...)
Prospective	Design	Simulate non-constant accrual over time (e.g., piecewise exponential), possibly varying with the number of treatments in the trial
Prospective	Design	Flexible cohort structures allowing for heterogeneity across treatments evaluated with potential biomarker enrichment in some cohorts, different control groups in other cohorts, the ability to evaluate for dose-response relative to the primary outcomes in other cohorts
Prospective	Design	There may be just two or three patient subgroups, it should be possible for treatments to fail or succeed in subgroups separately (i.e., be stopped with respect to one treatment but to carry on being assigned in another)
Prospective	Design	There may be many subgroups, in which case treatment stopping might be decided on the treatment’s performance in predefined “signatures”. Combinations of sub-groups that are medically consistent and a large enough sub-population to be clinically and commercially significant
Prospective	Design	If response adaptive randomization is being used in a trial with sub-groups it should be performed based on the treatment effect at the sub-group level
Prospective	Design	Allow treatments to have sub-arms (e.g., different doses, possibly with dose response models across the arms, and adaptive allocation between the arms)
Prospective	Design	Allow Treatments to have differing treatment duration within the trial: different subject allowance, different stopping rules
Prospective	Design	Allow treatments to be used in combination therapy. It maybe that treatments are only combined if they are from different treatment groups, it maybe that some treatments are only used in combination.
Prospective	Design	Allow participants to be re-randomized to new intervention after completing participation in another cohort within the platform trial
Prospective	Reporting	Simulation (and analysis) of longitudinal response trajectories for participants, along with reporting of patient-level simulation data

Summarizing both trajectories, widely applicable and usable software will require a common well-documented architecture decomposing the designs of platform studies into a number of modular functions, which are common to all packages and would then allow for customization by the user.

## Discussion

We have conducted a systematic literature search to identify commercial and open-source software aimed at designing platform and multi-arm and multi-stage clinical trials. Commercial and open-source software were differentiated and further software indirectly relevant to the design of such trials was recorded. A recently conducted software review focusing on adaptive designs is partially relevant in the context of platform trials [13]. Our systematic search was performed using Scopus and Web of Science Core Collection and augmented by the results of a recent systematic review on platform trials and other master protocol trials [3] and manual searches on online software repositories. This search strategy has limitations, such as only identifying software solutions, which either have a publication related to them or which can be found on GitHub/Rseek using certain keywords. Furthermore, the decision whether a particular piece of software is relevant in this context is to some degree subjective, making false negatives possible despite exercising utmost care during article screening and full-text assessment.

Currently, only a handful of software packages are aimed directly at simulating platform trials, while additional software packages exist with the goal of simulating multi-arm multi-stage (MAMS) designs. MAMS design software can be useful for platform trial design, since it can handle multiple treatments, a wide range of different types of endpoints and allow for one to control the family-wise type I error rate. However, many design elements specific to platform trial designs (e.g., staggered entry of treatments over time, probabilities of adding new treatments over time, complex decision at interim, etc., see Fig. 1) are usually not tackled by such software. Due to the near unlimited complexity of platform trials, no software will ever be able to be arbitrarily flexible and include all possible design options. Instead, for a particular software package, one needs to make a trade-off between developing a standalone software package which might not be used by other researchers in the future against incorporating new methods for existing modular software projects such as OCTOPUS. Developing a standalone simulation software package for a specific problem might have the advantage that less programming time is needed compared to extending already existing software packages, because for the latter familiarizing oneself with the code is sometimes time-consuming. Furthermore, it might not be foreseen in the existing software to add specific features, e.g., selection rules at interim, which makes implementing specific design features a difficult task. Therefore, building on an existing software only makes sense if it already provides enough modular structure and documentation to customize it to the specific objectives of a new platform trial.

Apart from the software mentioned in the “Results” section, the majority of the provided software is very specialized and only targets single trial design elements and none have the flexibility to handle a wide range of options required in many of the platform trials designed today and only the Integrated Platform for Designing Clinical Trials offer extensive options and different types of Bayesian platform trial designs where dynamic sharing has become commonplace. Furthermore, most software does not provide systematic and structured documentation such that it is nearly impossible for users to implement the software without analyzing the source code. This is important given that many clinical trialists often are not experts in the underlying programming language. Here, in addition to a high-quality documentation, a user-friendly graphical user interface (GUI) may be needed for the software’s broader application and setting up basic designs to build first discussions upon. For R packages, R markdown offers excellent options to provide clean and informative vignettes (http://r-pkgs.had.co.nz/vignettes.html). While many R packages provide such a vignette, most of them do not include a GUI. An example for a user-friendly, yet feature-packed Shiny App with a graphical user interface is multiarm. Accessible on https://mjgrayling.shinyapps.io/multiarm/ with the source code at https://github.com/mjg211/multiarm, this R Shiny app [30] facilitates the design and analysis of fixed-sample, multi-arm single-stage clinical trials, whereby a focus is laid on different options of multiplicity correction (e.g., Dunnett, Hochberg, Benjamini-Hochberg, and many others). The output includes an extensive structured summary of the trial design as well as a detailed summary of error rates and operating characteristics, together with readily usable tables and figures. Another example is the Shiny implementation of the rpact R package at https://rpact.shinyapps.io/public/. Along several output tabs and the option to export a report based on the simulation settings, this Shiny app shows users the underlying R code used to generate the outputs. This allows users to take their first steps with the software using an online graphical user interface and in case more control is required, the code can be copied and built upon locally. It should be mentioned that not only R and Stata packages were identified: We also came across a Python project called BATS (Bayesian Adaptive Trial Simulator), which, according to the publicly available documentation (https://github.com/Testispuncher/BATS-Bayesian-Adaptive-Trial-Simulator), is a feature rich, Bayesian, multi-arm multi-stage trial simulator. However, the most recent commits were made in 2016, the website is offline (http://usebats.org/bats (as of 01-02-2021)) and installation failed on our machines. Unfortunately, many simulation studies neither provided nor mentioned any software (code), while others claimed code was available at the personal webpages of the authors, many of these were no longer accessible. Ideally software should be made available only through dedicated online repositories such as CRAN and Github, where also the last time of commits is immediately visible. There is also a rich landscape of R packages aimed at parallel group trial simulations, of which we would like to highlight the Mediana package (https://gpaux.github.io/Mediana/), for which recently a Beta version extension to adaptive trials was uploaded on GitHub (https://github.com/medianainc/MedianaADT).

With the global pandemic that has evolved throughout 2020, the relevance of adaptive platform trials outside of oncology will become increasingly important in order to be able to successfully conduct drug development in both common and rare diseases [31–33]. As pointed out by one of the reviewers, the RECOVERY trial conducted in the UK proved how efficient platform trials can become in a pandemic situation. Key factors for RECOVERY’s success were a dedicated funding, strong commitment of NHS hospitals and a quick start with easy-to-use operation and protocols [34]. Especially funding raises a big challenge to initiate and run a platform trial in the first place. Public funding such as the NIHS funding for RECOVERY or EU Innovative Medicine Initiative funding for EPAD [35] seem to be promising models for the future to avoid that platform trials just remain a dream mainly for statisticians only, as innovative Bayesian clinical trials were once described [36]. Similarly, funding is also a challenge for software development projects and subsequent support and maintenance. The latter is usually included when purchasing commercial software, but is not guaranteed for open-source and free to use software solutions.

The modernization of analytics and the increases in regulatory requirements has created challenge for those to trying to find the best statistical software package for application in the development of the best platform trial design. This review has highlighted the many gaps that exist in the available software packages that are currently being applied in different settings. The potential reason for these gaps is lack of a readily available framework that allows for the development, maintenance, and continued support of these statistical software packages. The support to establish such a framework is critical such that when open source statistical software packages become available and can be accessed through Github, CRAN, and other platforms, the user will have an algorithm by which they can determine whether or not the statistical software package they are using will be able to reliably generate reproducible results in support of their platform trial design scenarios. Thus, there is still room to grow in the development of a platform trials software package that can handle the core requirements, with the possibility of modular expansion to be able to handle a wider of variety of platform trial designs over time.

## Supplementary Information

**Additional file 1: S1** Spreadsheet containing information about all the screened articles and extracted information.

**Additional file 2: S2** PRISMA Checklist

**Additional file 3: S3** Further information regarding definition of features in Table 2.

## Data Availability

All data generated or analyzed during this study are included in this published article.
